# Direct link between convergent evolution at sequence level and phenotypic level of septal pore cap in Agaricomycotina

**DOI:** 10.1093/g3journal/jkag176

**Published:** 2026-07-06

**Authors:** Tomoyo Iizuka, Masafumi Nozawa, Kazuho Ikeo

**Affiliations:** Department of Genomics and Evolutionary Biology, National Institute of Genetics, 1111 Yata, Mishima, Shizuoka 411-8540, Japan; Department of Genetics, SOKENDAI, 1111 Yata, Mishima, Shizuoka 411-8540, Japan; Department of Biological Sciences, Tokyo Metropolitan University, 1-1 Minami-Osawa, Hachioji, Tokyo 192-0397, Japan; Research Center for Genomics and Bioinformatics, Tokyo Metropolitan University, 1-1 Minami-Osawa, Hachioji, Tokyo 192-0397, Japan; Department of Genomics and Evolutionary Biology, National Institute of Genetics, 1111 Yata, Mishima, Shizuoka 411-8540, Japan; Department of Genetics, SOKENDAI, 1111 Yata, Mishima, Shizuoka 411-8540, Japan

**Keywords:** convergent evolution, morphological evolution, comparative genome analysis, fungi, septal pore cap

## Abstract

Several homologous morphological characters, despite sharing apparently similar features, are known to have independently evolved in different lineages multiple times. However, the genetic backgrounds of such morphological convergences remain poorly understood. To detect any correlated amino acid substitutions potentially responsible for morphological convergence at the phenotypic level, we focused on the morphology of the septal pore cap (SPC), a structure involved in mycelia's complex multicellularity in fungi. SPCs are classified into 3 morphological types: perforate, imperforate, and vesiculate. To understand the evolutionary events that occurred at the sequence level during the morphological convergence of perforate SPCs in Agaricomycotina, we examined sequence differences among species with different SPC types by comparative genomic analysis using a single-copy gene dataset from 12 Agaricomycotina genomes with morphological literature of SPC. Our analysis revealed that sequences of 8 genes, including an SPC-related gene *spc33*, were clustered based on SPC morphology rather than species relationship. Additionally, same amino acid substitutions independently occurred in both lineages in which species with perforate SPCs emerged. These findings suggest that specific amino acid substitutions in *spc33* were critical for the emergence of perforate SPCs in multiple lineages. Further, our gene search for *spc33* across organisms suggests that *spc33* evolved shortly before the emergence of imperforate SPC. This study represents the first step toward elucidating the genetic basis of the morphological evolution of SPC. It contributes to both clarifying the genetic basis underlying morphological convergence and advances the study of fungal evolutionary morphology.

## Introduction

An important problem in evolutionary biology is determining what changes at the genome sequence level have contributed to morphological diversity. The study of convergent evolution, ie the independent evolution of similar morphological changes in different lineages, is thought to provide insight into hotspots of key mutations for phenotypic differentiation (Stern and Orgogozo 2009). In addition, focusing on morphological convergence provides clues for understanding the relationship between phenotypic evolution affected by natural selection and sequence evolution that can be described by the protein adaptive landscape (Castoe et al. 2010; Stern 2013; Almén et al. 2016). Therefore, understanding convergent evolution at the sequence level is also crucial for identifying the substitutions responsible for convergent evolution at the phenotypic level. However, genome-wide surveys of convergent evolution remain limited, and more empirical genome-scale studies are needed to elucidate the genetic basis of morphological convergence.

**Fig. 1.**
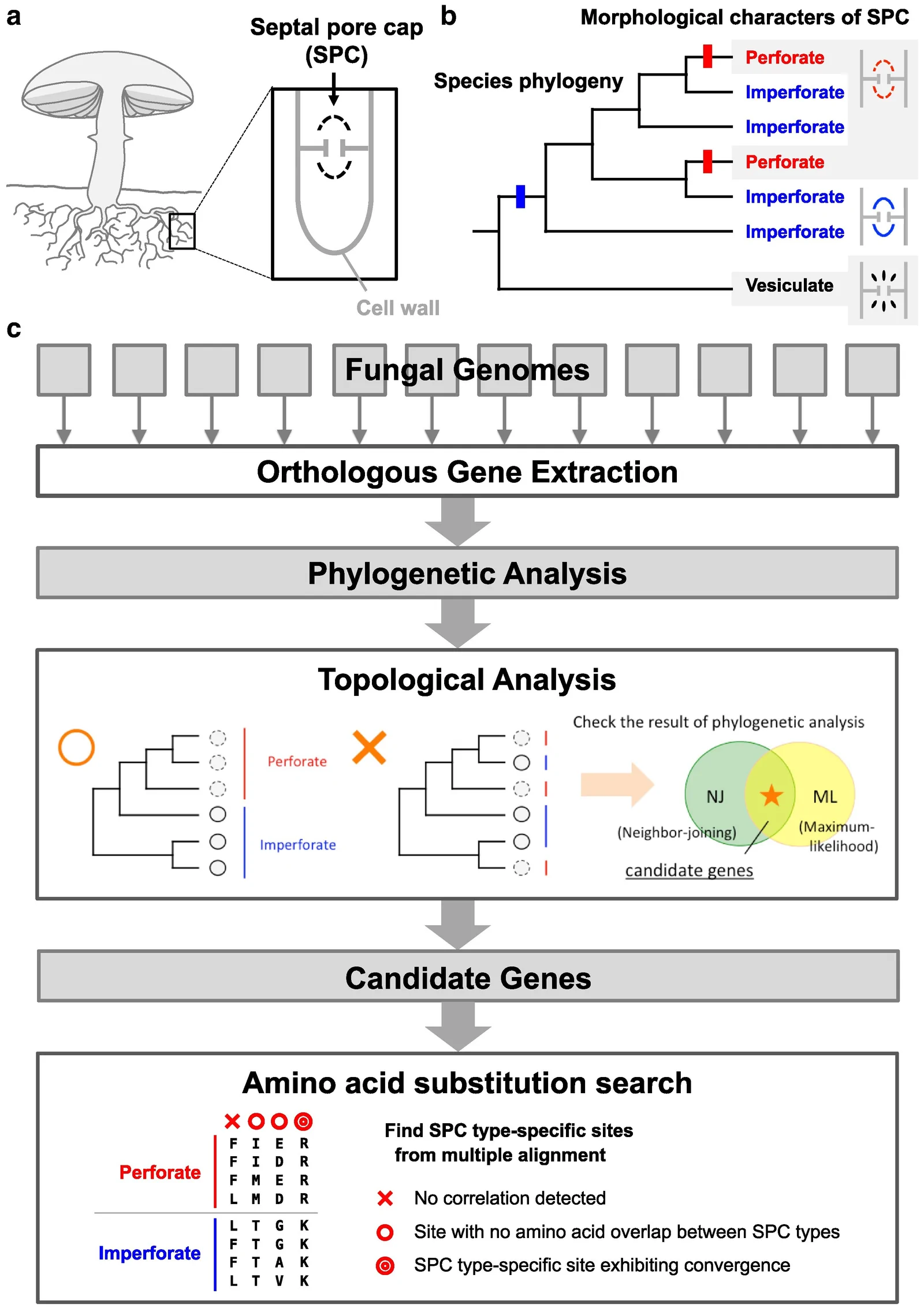
Study design used to identify candidate genes and convergent amino acid substitutions associated with septal pore cap morphology. a) Cellular location of the septal pore cap (SPC), a structure associated with septation in fungal hyphae. b) Distribution of perforate, imperforate, and vesiculate SPC types on a species phylogeny, showing that perforate SPCs evolved multiple times independently. c) Schematic overview of the candidate gene extraction pipeline. Orthologous genes were first identified, and gene trees were constructed for each ortholog. In the topological analysis step, gene trees were evaluated based on whether a branch clearly separated perforate and imperforate SPC species. Circles indicate gene trees whose topology was consistent with SPC morphology (ie showing a clear split between perforate and imperforate groups), whereas crosses indicate gene trees that did not meet this criterion and were excluded from further analyses. Genes passing this topological filter were subsequently subjected to the amino acid substitution search step to detect convergent sites using both the traditional approach and the PCOC method.

One such example of morphologically convergent traits is fungal septal pore caps (SPCs), which maintain cytoplasmic continuity between adjacent fungal hyphal compartments by modulating septal pore permeability, allowing selective cytoplasmic exchange while restricting organelle passage and sealing the pore upon hyphal damage to maintain mycelial integrity (Jedd 2011; Nguyen et al. 2017; Nagy et al. 2020) (Fig. 1a). It was suggested that SPCs were newly derived from the endoplasmic reticulum (ER), as evidenced by the staining reaction of SPCs with specific ER-targeting markers (Müller et al. 1999; van Driel et al. 2008), and the structural connection between the base of SPCs and the ER (Moore 1975; Müller et al. 1998a, 2000). Thus, understanding the genetic basis of SPC provides fundamental insight into the origin of multicellularization in fungi and the mechanism of organelle specialization of SPC.

We investigated 3 morphological types: vesiculate, imperforate, and perforate SPC, which differ primarily in whether the SPC itself contains a perforation and in the associated membranous structures (Fig. 1b). In Agaricomycotina, taxa of the most ancestral lineage have vesiculate SPCs, whereas taxa of all other lineages have either imperforate or perforate SPCs. A previous phylogenetic study showed that perforate SPCs independently evolved from imperforate SPCs multiple times (van Driel et al. 2009); therefore, perforate SPCs evolved under morphological convergence. The different SPC types result in different cytoplasmic transport functions inside the mycelium (Bracker and Butler 1964; Moore and Marchant 1972), which indicates that SPC types evolved through adaptation to transport various substances. Additionally, the feature of SPC types being highly conserved at the order level has played a key role in morphological observations within taxonomy (Hibbett 2006; van Driel et al. 2009; Oberwinkler et al. 2013; Hibbett et al. 2014). This highlights that unraveling the genetic basis of SPC morphological differentiation can significantly contribute to understanding the evolution of higher taxa in Basidiomycota.

Previous SPC studies reported on their functions, fine features, and applicability to fungal classification (Bracker and Butler 1964; Moore and Marchant 1972; Lisker et al. 1975; Patton and Marchant 1978; Hibbett 2006; van Driel et al. 2009); however, despite the long history of such morphological studies on SPCs, the genetic basis of morphological differentiation in SPC types remains unclear. In accordance with established techniques, most studies focused on phenotypes larger than the nanoscopic scale when reporting substitutions associated with morphological convergence by whole-genome analysis. For example, Hu et al. (2017) found a candidate gene for the evolution of a pseudo-thumb in pandas using positive selection as an indicator. Colosimo et al. (2005) found that the Eda pathway is involved in the phenotypic convergence of armor plates in sticklebacks. These findings motivated us to identify the key genomic changes associated with morphological convergence of SPC, that is, hard to tackle using reverse or forward genetics approaches using genomic approaches.

In this study, we searched signatures of sequence evolution that had evolved with morphological convergence of SPC by comparative genomics. We collected publicly available whole-genome sequences from the Joint Genome Institute portal (JGI, https://genome.jgi.doe.gov/portal/). Data on morphological types of SPC were obtained from previous studies, which were accumulated due to their significance in taxonomic classification (Wells 1994; Müller et al. 1998b, 2000; Lutzoni et al. 2004; Hibbett et al. 2014). Although the available pairs of genomic and morphological data were limited, we successfully inferred evolutionary events that corresponded to morphological convergence at the amino acid sequence level using datasets of the representative 12 Agaricomycotina species. Here, we report that convergent evolution had occurred in sequence level as well as phenotypic level. Our results uncover key amino acid substitutions directly correlated with the morphological convergent evolution from imperforate to perforate SPCs.

## Materials and methods

### Genome sequences

The coding sequence data for 12 species of Basidiomycota, which included 11 representatives of Agaricomycetes (6 perforate species and 5 imperforate species) and 1 representative of Dacrymycetes (imperforate type), were used for this study (Table 1). These species are representative of major Basidiomycota species. All sequence data were obtained from the JGI of the United States Department of Energy.

**Table 1.** List of fungal taxonomic classifications, SPC types, and genomic data sources from JGI (http://genome.jgi.doe.gov/programs/fungi/index.jsf).

Class	Order	Species	SPC type (van Driel et al. 2009)	Genome assembly size (Mbp)	No. of Genes	JGI abbreviation	Genome reference
Agaricomycetes	Agaricales	*Galerina marginata*	Perforate	59.42	21,451	Galma1	Riley et al. 2014
	Corticiales	*Punctularia strigosozonata*	Perforate	34.17	11,538	Punst1	Floudas et al. 2012
	Gloeophyllales	*Gloeophyllum trabeum*	Perforate	37.18	11,846	Glotr1	Floudas et al. 2012
	Polyporales	*Phanerochaete carnosa*	Perforate	46.29	13,937	Phaca1	Suzuki et al. 2012
	Hymenochaetales	*Rickenella mellea*	Perforate	46.03	18,935	Ricme1	Nagy et al. 2016
		*Trichaptum abietinum*	Imperforate	40.61	14,963	Triab1	(Project ID: 1,006,923)
	Trechisporales	*Sistotremastrum niveocremeum*	Imperforate	35.36	13,069	Sisni1	Nagy et al. 2016
	Gomphales	*Ramaria rubella*	Imperforate	105.46	19,264	Ramac1	(Project ID: 1,016,735)
	Cantharellales	*Rhizoctonia solani* AG-1 IB	Perforate	47.66	16,497	Rhiso1	Wibberg et al. 2013
		*Tulasnella calospora*	Imperforate	62.39	19,535	Tulca1	Kohler et al. 2015
		*Botryobasidium botryosum*	Imperforate	46.67	15,034	Botbo1	Riley et al. 2014
Dacrymycetes	Dacrymycetales	*Calocera viscosa*	Imperforate	29.10	12,369	Calvi1	Nagy et al. 2016

### Ortholog identification

Orthologous genes were identified using the reciprocal best hit (RBH) approach based on all-vs-all BLASTP comparisons (Tatusov et al. 1997; Bork et al. 1998; Overbeek et al. 1999). This conservative RBH-based approach restricted the dataset to orthologous groups with reciprocal best-hit relationships across all 12 species, thereby obtaining high-confidence one-to-one orthologs while minimizing paralog inclusion and other orthology misassignments. We note that RBH may underestimate orthology in the presence of gene duplication or deep sequence divergence, but provides a robust and reproducible framework for the analyses conducted here. We performed all-vs-all comparison with BLAST software (blastp, NCBI-blast-2.2.30+) using the coding sequences that remained after discarding sequences with internal stop codons. The analysis was conducted using the default parameters of the blastp program. The RBH, genes with the best hits to each other in each of the 11 comparisons, were extracted using original Perl scripts. Scripts used in this study are available at https://github.com/toiizuka/SPC-convergence-pipeline.

### Phylogenetic analysis of orthologous gene dataset

To extract candidate genes associated with the morphological differences between imperforate and perforate SPCs, we conducted phylogenetic analyses on orthologous genes and evaluated their topologies. The multiple alignment was constructed using MUSCLE v3.8.31 (Edgar, 2004). Phylogenetic gene trees for each ortholog were reconstructed using both the neighbor-joining (NJ) (Saitou and Nei 1987) and maximum-likelihood (ML) methods (Felsenstein 1981) implemented in MEGA6-CC (Tamura et al. 2013); for ML analyses, the best-fit substitution model was selected using MEGA6-CC. We then checked the topology of the trees. To confirm the phylogenetic relationships of the 12 fungal species, a concatenated tree was constructed utilizing a dataset of all RBH orthologs. The NJ method was used for this purpose. We also confirmed the reliability of each gene tree by conducting the approximately unbiased (AU) test (Shimodaira 2002) with IQ-TREE v1.16.12 (Chernomor et al. 2016). This AU test assessed the confidence of tree selection comparing the topology of the resulting gene tree with 2 types of topologies: species phylogeny and the topology of the obtained gene tree (clustered by SPC morphology). Accordingly, we used the AU test to assess whether the topology of each gene tree could be statistically rejected based on its sequence data, thereby providing a supplementary check on the plausibility of inferred gene tree topologies. In the topological analysis, a gene was regarded as a candidate for convergent evolution when all genes from species with perforate SPCs were grouped within the same clade in both NJ and ML trees, which were constructed as unrooted (circle in Fig. 1c, “Topological analysis”). This unrooted approach was used to avoid bias introduced by uncertain rooting, and the classification was determined by whether a branch clearly split the perforate and imperforate SPC groups. Notably, when the trees were alternatively rooted based on the inferred species phylogeny (with *Calocera viscosa* [imperforate] as the most plausible species for rooting), the perforate SPC genes still formed a monophyletic clade, supporting the robustness of this criterion. The obtained genes were annotated using BLAST (Johnson et al. 2008) against 5 databases, NCBI nonredundant protein sequences, UniProt (259, 2017-04) (The UniProt Consortium 2017), Gene Ontology (2017-04-11) (Ashburner et al. 2000), *Saccharomyces* Genome Database (R64.2.1, 2014-11-18) (Cherry et al. 2012), and PomBase (30_62, 2017-01-30) (Wood et al. 2012).

### Detection of key amino acid substitutions for morphological convergence

To extract key sites for morphological convergence at the single amino acid site level from multiple alignments of candidate genes constructed in the “Phylogenetic analysis of orthologous gene dataset” section, we first applied a traditional method based on ancestral sequence reconstruction. Ancestral amino acid sequences for each ortholog were reconstructed using the ML algorithm implemented in MEGA6-CC (Tamura et al. 2013). Next, amino acid substitutions were mapped onto branches associated with transitions in SPC morphology. Candidate convergent sites were selected where the same amino acid state independently arose in multiple lineages exhibiting the same SPC type. We additionally checked whether extant sequences showed distinct amino acid states between perforate and imperforate species, with no overlap in observed amino acid states (circle and double circle in Fig. 1c, “Amino acid substitution search”). This approach follows the general framework of previous studies (eg Zhang and Kumar 1997), but differs in that we did not perform a formal statistical test to evaluate whether the observed number of convergent substitutions exceeds that expected under a neutral model. Instead, this method serves as a heuristic approach to identify candidate sites showing repeated, phenotype-associated substitutions across independent lineages. All substitutions were extracted using a custom Perl script based on the reconstructed ancestral sequences. The script and associated data are provided in the “Data availability” section.

We additionally employed Profile Change with One Change (PCOC) v.1.1.0 (Rey et al. 2018), a method for detecting convergent substitutions and shifts in site-specific amino acid (Rey et al. 2018). This analysis was performed using the default settings with a posterior probability >0.8 (option -f 0.8). Only sites identified by the PCOC model were extracted. CSUBST v.1.11.10 (Fukushima and Pollock 2023) and evolutionary sparse learning with paired species contrast (ESL-PSC) v.2.1.0 (Allard et al. 2025) were also performed as complementary analyses. ESL-PSC analysis was conducted using all 2,560 orthologs as input to evaluate whether the 8 candidate genes exhibit a higher frequency of convergent signals compared to the genomic background. For this analysis, the command options –use_logspace and –cancel_only_partner were specified, and species groups were defined as follows: (Punst, Glotr, Phaca, Galma, Ricme), (Triab, Sisni, Ramac), (Rhiso), and (Tulca, Botbo, Calvi). CSUBST was applied to identify convergent sites at the codon level within the candidate genes.

To evaluate alternative explanations for the observed convergent substitutions, including horizontal gene transfer (HGT), local synteny surrounding *spc33* and exon–intron structures of *spc33* were examined across 12 species. Conservation of genomic context and gene structure was used to assess vertical inheritance of this locus.

## Results

### Phylogenetic gene trees and candidate gene extraction

**Fig. 2.**
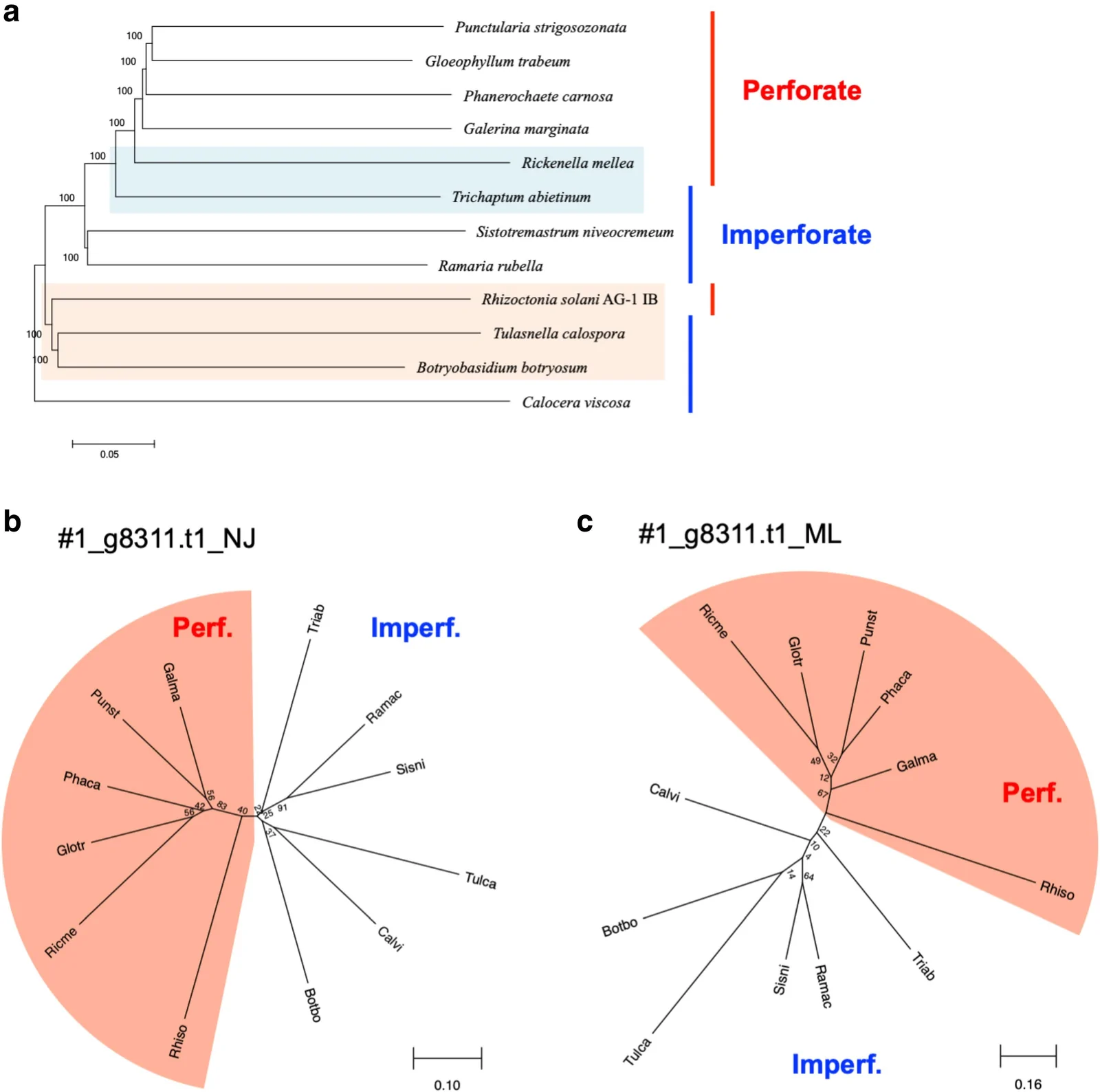
Phylogenetic relationship of 12 fungal genomes and correlated genes. a) Phylogenetic tree based on the neighbor-joining method of concatenated sequences of 2,560 orthologs. Numbers near internodes correspond to values of 1,000 bootstrap replicates. *Hymenochaetales* (red) and *Cantharellales* (blue) were used for multiple species. b) and c) Gene trees of the correlated gene #1 based on b) the neighbor-joining and c) the maximum-likelihood methods. Phylogenetic gene trees of correlated genes #2–#8 were compiled in Supplementary Fig. 1. The abbreviations of the species names are as follows: Punst, *Punctularia strigosozonata*; Glotr, *Gloeophyllum trabeum*; Phaca, *Phanerochaete carnosa*; Galma, *Galerina marginata*; Ricme, *Rickenella mellea*; Triab, *Trichaptum abietinum*; Sisni, *Sistotremastrum niveocremeum*; Ramac, *Ramaria rubella*; Rhiso, *Rhizoctonia solani* AG-1 IB; Tulca, *Tulasnella calospora*; Botbo, *Botryobasidium botryosum*; Calvi, *Calocera viscosa*. Scale bar at the bottom of each tree indicates the branch length.

The RBH analysis yielded 2,560 single-copy orthologs. Using these orthologs, we constructed concatenated sequence tree to infer species phylogeny and gene trees to test each ortholog. The resulting concatenated tree (Fig. 2a) was consistent with the currently accepted species phylogeny (He et al. 2024) and was therefore used for subsequent analyses. Gene trees constructed with the NJ method (Fig. 2b) and ML method (Fig. 2c) showed that 8 genes of the perforate type species *Rhizoctonia solani* AG-1 IB (Rhiso1 in JGI; Wibberg et al. 2013) clustered with those of other perforate type species from another lineage (Fig. 2, Table 2, Supplementary Fig. 1). Our functional prediction assigned functional information for 7 of the 8 genes (Table 2). Although the group of candidate genes includes a wide range of functions, interestingly, the gene g8311.t1 was annotated as SPC-related gene, *spc33* (van Peer et al. 2010). We further confirmed with the AU test that none of the extracted gene tree topologies were rejected (Supplementary Table 1). For example, the *P*-value of the AU test for *spc33* was 0.49 with the appropriate amino acid substitution model for the *spc33* gene tree. Therefore, the result of phylogenetic analysis shows clear pattern of topological clustering by SPC morphology.

**Table 2.** List of 8 candidate genes.

	Gene ID	BS NJ*^a^*	BS ML*^a^*	Model*^b^*	aa*^c^*	Gene symbol	Full gene name
#1	g8311.t1	40	22	LG + G	596	*spc33*	33 kDa protein purified from SPCs
#2	g3005.t1	12	10	LG + G	468	DSD1	D-serine dehydratase
#3	g3493.t1	40	46	rtREV + G	103	SDH8	Succinate dehydrogenase
#4	g7563.t1	16	12	LG + G	318	n/a	Uncharacterized protein
#5	g6611.t1	10	10	LG + G	98	SUMO	Small ubiquitin-like modifier protein
#6	g5937.t1	38	38	JTT + G + I	454	ELP4	Elongator acetyltransferase complex subunit 4
#7	g1797.t1	10	13	LG + G + I	271	DRE2	Fe-S cluster assembly protein
#8	g14383.t1	21	13	LG + G	221	DUS1	tRNA-dihydrouridine synthase 1

Gene ID and amino acid length were referred from the CDS file of public JGI data source of *Rhizoctonia solani* AG-1 IB (Rhiso).

^*a*^Bootstrap (BS) value of the neighbor-joining (NJ)/maximum-likelihood (ML) tree supporting a single perforate SPC cluster.

^*b*^The best-fit substitution model of the ML tree.

^*c*^Amino acid length.

### Candidate genes under convergent evolution in the emergence of perforate SPC

The detection of significant topology linking morphological convergence from gene trees motivated us to explore the presence of convergence at the amino acid site level, which can be marked as an independent change to the same amino acid in the lineage to perforate type species. From 2,560 single-copy orthologs, 60,131 and 2,020 convergent sites were detected by PCOC analysis and our traditional method, respectively (Supplementary Fig. 2). 72.5% of the sites detected by the traditional method were also identified as the convergent sites by the PCOC method. Although PCOC detected a much larger number of sites due to the permissive threshold applied, most sites identified by the traditional method were also recovered by PCOC; accordingly, unambiguous convergent sites—defined as amino acid substitutions independently detected by both approaches—were observed in 4 of the 8 candidate genes (g8311.t1 [*spc33*], g6611.t1 [SUMO], g5937.t1 [ELP4], and g1797.t1 [DRE2]), whereas the remaining genes showed no overlap between methods (Supplementary Table 2). Among genes with convergent substitutions, the number of convergent sites detected by PCOC method increased proportionally with the total number of substitutions in the ortholog (*R* = 0.94) (Supplementary Fig. 3a). This means that PCOC detects more convergent substitutions from genes with higher substitution rates. Furthermore, the correlation between the number of PCOC-detected sites and total number of substitutions was stronger than that between the number of PCOC-detected sites and gene length (Supplementary Fig. 3b). This indicates that, contrary to the intuitive expectation that longer genes would naturally accumulate more substitutions, convergent substitutions do not increase proportionally with gene length. We then compared the number of convergent substitutions per gene length identified by the PCOC approach with that identified by the traditional approach. This comparison revealed no clear correlation between the 2 methods (Supplementary Fig. 3c), suggesting that they rely on fundamentally different strategies for detecting convergence. Since our method detects only amino acid sites that exhibit residues clearly distinguishable depending on morphological characters, the sites identified by both methods are considered to be more reliable convergent sites. The results of these 2 analyses indicate that only a small number of sites correspond to the morphological convergence of SPC.

### Comparison of convergent substitution accumulation

**Fig. 3.**
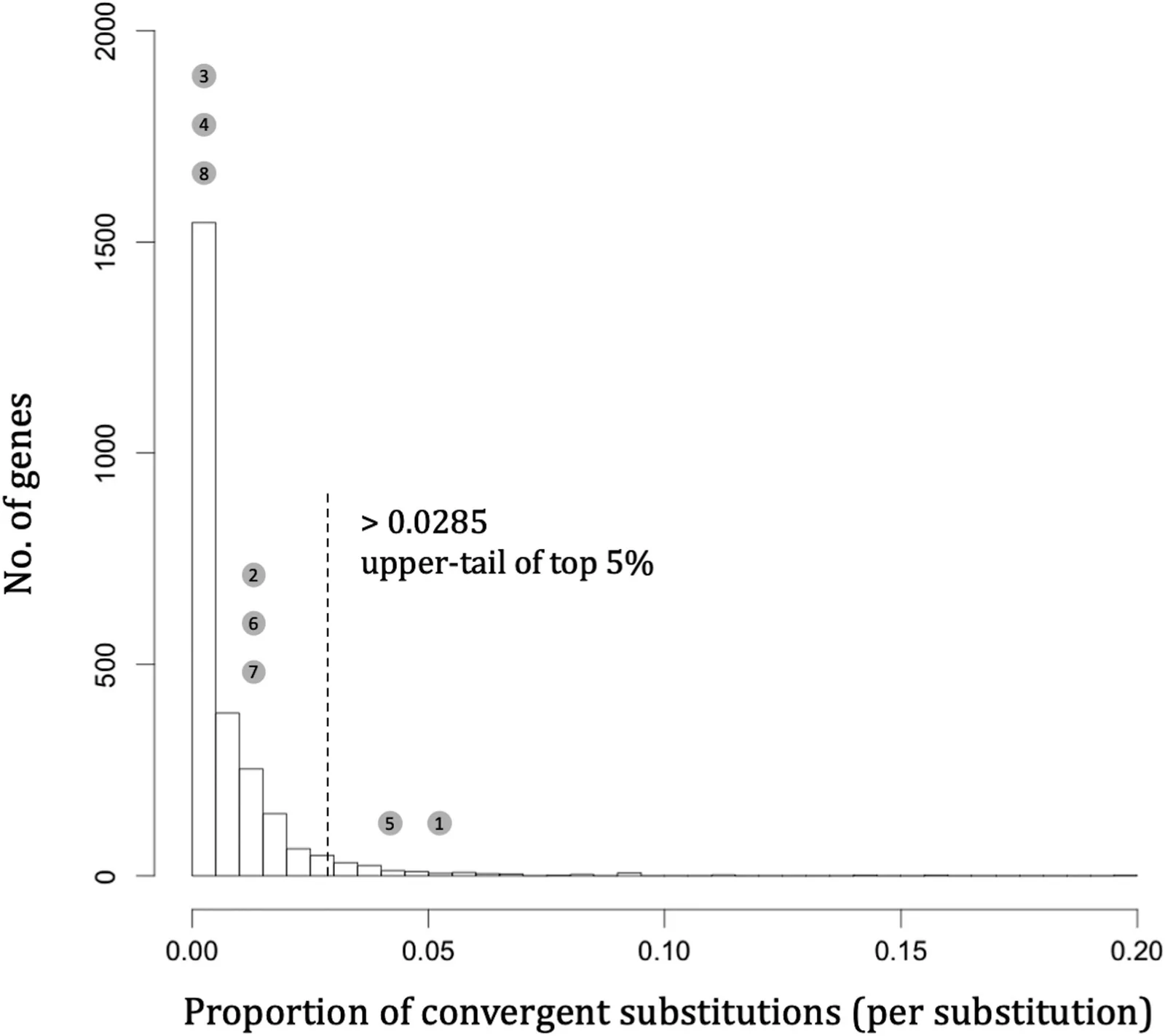
Frequency distribution of convergent substitution rates across 2,560 genes. The *x* axis shows the proportion of convergent substitutions as described at 2.3. This showed that many genes do not have convergent substitutions. Numbers 1 through 8 correspond to those in Table 2. A substitution was regarded as a convergent substitution if an amino acid independently changed to the same amino acid in the lineage to perforate species. This “number of convergent substitutions” was calculated for each ortholog. Then, we calculated the number of convergent substitutions per whole substitutions for each ortholog; for example, when an ortholog carries 3 convergent substitutions with the total number of substitutions of 100, it becomes 0.03 (3/100).

To test whether the robust convergent substitutions shown in the previous step were accumulated in each gene, we checked the number of convergent substitutions detected by the traditional method in all genes of Rhiso and other perforate lineages and then compared the frequency of convergent substitutions among orthologs. Among the 2,560 genes, 1,416 genes included in the smallest bin (1,546 genes) did not contain any convergent substitutions at their amino acid sites (Fig. 3). This highlights that convergent substitutions are rare within the gene dataset. We also confirmed that 4 of the candidate genes ranked within the top 1% and 3 within the top 1.5%, indicating that convergent signals in our candidate genes occur more frequently than in other genes (Supplementary Table 3). In addition, we found that convergent substitutions were accumulated in the gene g8311.t1 (*spc33*). The results showed that the proportion of convergent substitutions for *spc33* was 0.055, which was top 1.3 percentile among all genes analyzed (Fig. 3). Another candidate gene, g6611.t1, was positioned in the top 5% percentile. However, unlike *spc33*, not all of the other 7 genes carried many convergent substitutions. These findings indicate that *spc33* has a higher convergent substitution rate than other orthologs.

### Candidate amino acid substitutions under convergent evolution in *spc33*

**Fig. 4.**
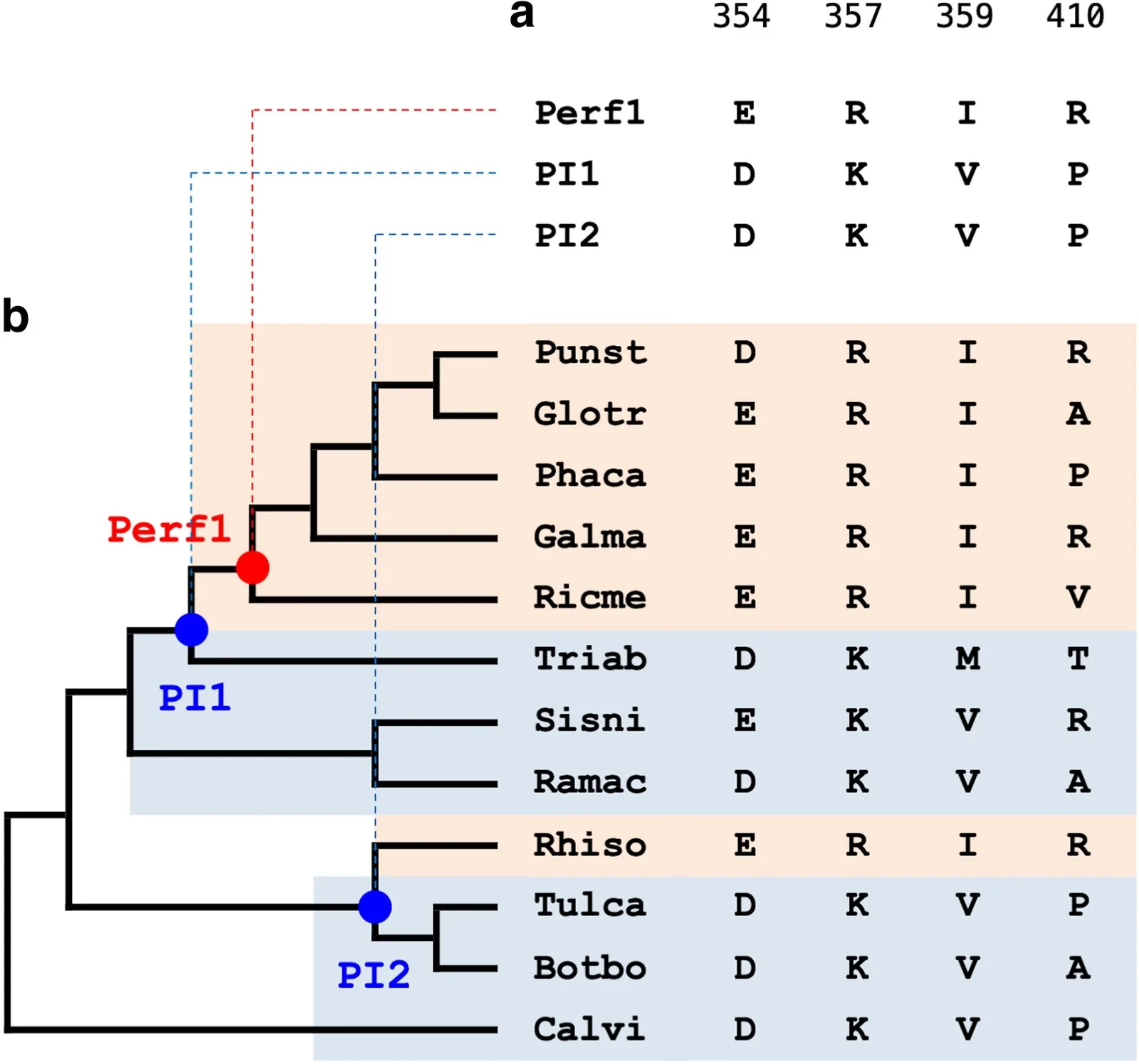
SPC type-specific convergent substitutions of *spc33* in perforate type species (red background) and imperforate type species (blue background). a) Ancestral sequences of focal sites. The abbreviations of the branch names are as follows: Perf1, lineage including 5 perforate species; PI1, perforate–imperforate branching node 1; PI2, perforate–imperforate branching node 2. b) SPC type-specific amino acid residues in extant species. The abbreviations of the species names are as follows: Punst, *Punctularia strigosozonata*; Glotr, *Gloeophyllum trabeum*; Phaca, *Phanerochaete carnosa*; Galma, *Galerina marginata*; Ricme, *Rickenella mellea*; Triab, *Trichaptum abietinum*; Sisni, *Sistotremastrum niveocremeum*; Ramac, *Ramaria rubella*; Rhiso, *Rhizoctonia solani* AG-1 IB; Tulca, *Tulasnella calospora*; Botbo, *Botryobasidium botryosum*; Calvi, *Calocera viscosa*.

We expected that if convergent evolution had occurred not only at the phenotypic level but also at the amino acid sequence level, the phenotypic characters of perforate SPCs may be regulated under a limited combination of molecular pathways. To investigate this possibility, we searched for candidate amino acid substitutions under convergent evolution in ancestral states of *spc33* from imperforate to perforate species. The results showed the same pattern of convergent amino acid substitutions at D354E, K357R, V359I, and P410R during evolution from perforate–imperforate branching point 1 (PI1) to the lineage including 5 perforate species (Perf1) and from perforate–imperforate branching point 2 (PI2) to Rhiso (*R. solani* AG-1 IB) (Fig. 4). Interestingly, 3 of the 4 sites were localized within the same short region consisting of 17 amino acids, even though SPC33 is more than 300 amino acids in length. This finding suggests that sequence differences in this region of *spc33* may be associated with the morphological convergence of perforate SPCs, although further experimental validation is required to confirm causality. At the substitution K357R of *spc33*, both ancestral branching points PI1 and PI2 showed the same amino acid residue, lysine (Fig. 4a); alternatively, the estimated ancestral residue of site 357 was arginine at the ancestral branching point Perf1 (Fig. 4a), and the residue of Rhiso also changed to arginine at the emergence of this species (Fig. 4b). Also, this substitution pattern K357R from imperforate to perforate species was completely conserved in all fungal species (Fig. 4a). Therefore, the same amino acid substitution from lysine to arginine independently occurred when perforate species emerged. In other words, K357R and M/V359I still showed differences in the extant species (Fig. 4b, Supplementary Fig. 4); therefore, convergent evolution also occurred at the sequence level. Substitutions at D354E and P410R were also detected; however, these substitution patterns were not conserved in the extant species.

**Fig. 5.**
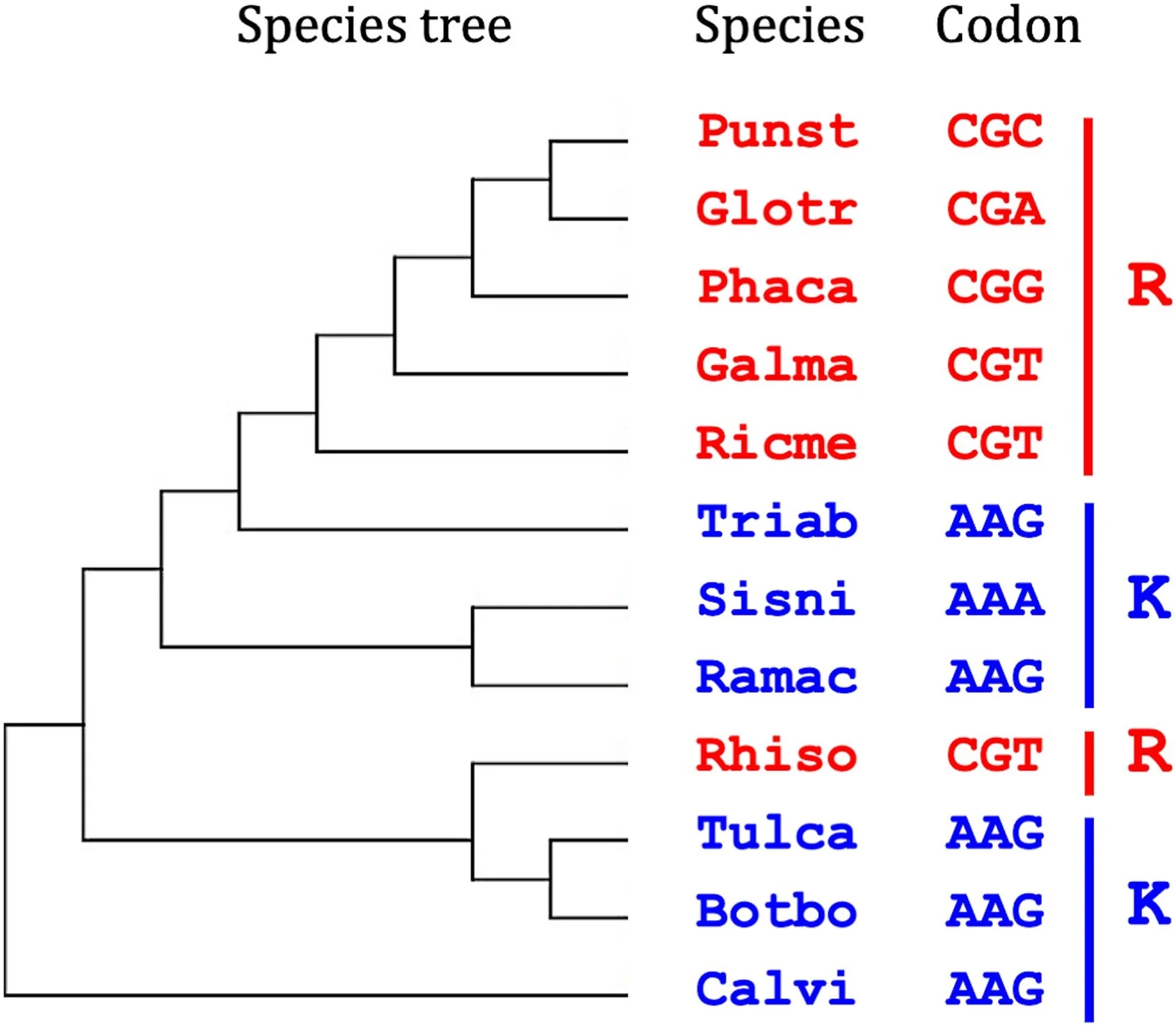
Codon sequences corresponding to SPC type-specific amino acid site 357 in *spc33*.

To understand substitution patterns in nucleotide sequences that resulted in amino acid substitution, we also checked site 357 at the nucleotide sequence level of both perforate lineages. Rhiso acquired the codon CGT (arginine) at site 357 (Fig. 5), and the 2 closest imperforate species (Tulca; *Tulasnella calospora* and Botbo; *Botryobasidium botryosum*) and the most basal species (Calvi; *Calocera viscosa*) use AAG (lysine). Other perforate species also use CG at the first and second codons. This result indicates that the substitution pattern of all perforate species has changed to CGN (arginine) from AAR (lysine). Thus, even at the nucleotide sequence level, we observed the same substitution pattern from AAG to CGT (Fig. 5). This convergent signal was also detected by CSUBST, which identifies convergence based on codon substitutions (Supplementary Table 4), further supporting *spc33* as a robust candidate gene.

To evaluate HGT as an alternative hypothesis for the observed convergent substitutions in *spc33*, we examined both local genomic synteny and exon–intron organization. Synteny analysis revealed that multiple orthologous genes are present in the genomic regions flanking *spc33* across species, indicating that the genomic position of *spc33* is conserved among lineages (Supplementary Fig. 5). Such conservation of local genomic context is inconsistent with recent horizontal transfer and does not support HGT as an explanation for the observed substitution patterns. In addition, analysis of exon–intron organization showed that *spc33* retains introns in Rhiso (*R. solani* AG-1 IB), arguing against retrotransposon- or virus-mediated horizontal transfer (Supplementary Fig. 6). Furthermore, the number and length of exons and introns were conserved among closely related species, suggesting that the exon–intron structure of *spc33* was established once and maintained through vertical inheritance. Together, these observations do not support HGT and instead are consistent with lineage-specific sequence evolution following morphological divergence.

### Feature of *spc33* in taxa other than perforate and imperforate type species

To compare *spc33* sequences with other organisms, we searched for *spc33* homologs against whole-sequence data available in the NCBI database and 41 fungal genome sequences in JGI, including the 12 fungal genomes tested in this study and genomes of species with vesiculate SPCs. The blastp results for *spc33* from *Calocera viscosa* (gene 532853) with an E-value cutoff of 1E-5 showed that *spc33* was not found in any vesiculate species, whereas *spc33* was found in all imperforate and perforate species (Table 3). Moreover, *spc33* homologs were not found in organisms other than perforate and imperforate species (including other fungi, other eukaryotes, and prokaryotes). These presence/absence patterns of *spc33* indicate that *spc33* emerged before the divergence of imperforate SPCs from vesiculate SPCs.

**Table 3.** Presence/absence pattern of *spc33*.

	Vesiculate (2)	Imperforate (11)	Perforate (28)
Presence (%)	0	100	100

Numbers in parentheses indicate the number of fungal species tested.

## Discussion

Van Driel et al. (2009) provided evidence for morphological convergence of SPCs based on species phylogeny. Here, we provide evidence for sequence convergence accompanying the morphological convergence of perforate SPCs. A substantial number of genome-wide studies have shown that when there is convergent evolution at the phenotypic level, different genes or different sites of the same gene would lead to the same phenotype (eg Hoekstra and Nachman 2003; Foote et al. 2015; Zou and Zhang 2015; Corbett-Detig et al. 2020). In other words, there are multiple paths that result in convergent phenotypic evolution. On the other hand, it was proposed that concentration of convergent amino acid substitutions at the same sites in the same genes that potentially contribute to phenotypic convergent evolution (eg Nei et al. 1997; Shi and Yokoyama 2003; Natarajan et al. 2016; Hague et al. 2017; Hu et al. 2017; Fukushima and Pollock 2023). This means a few convergent substitutions can cause phenotypic convergence. Our findings support the latter scenario regarding the convergent evolution of perforate SPCs. Although HGT represents a possible alternative scenario for the genetic basis of morphological evolution, the conserved local synteny, exon–intron organization, and stringent RBH-based ortholog selection provide no evidence supporting HGT or orthology misassignment as explanations for the observed convergence signals. Below, we discuss convergent evolution in fungal genome sequences and suggest the possible roles of the candidate genes detected (especially *spc33*, the gene identified as SPC-related genes by van Peer et al. 2010) in the morphological convergence of SPCs.

Phylogenetic and topological analysis against orthologous gene dataset identified the genes for which tree topologies are different from the topology of the species phylogeny. Although some convergent substitutions may occur at individual sites without affecting overall gene tree topology, our analyses indicate that genes showing topology shifts represent cases with particularly strong phenotype-associated signals. Among them, 8 genes exhibited clustering patterns of tree topology with a cluster of perforate SPC (Fig. 2b and c), implying a potential role of these genes in SPC type differentiation. Bootstrap support for individual gene trees of the 8 candidate genes is generally low. This is expected in genes where only a limited subset of sites is associated with phenotypic convergence, while the majority of the sequence evolves according to species divergence. Consequently, weak support for alternative topologies reflects the localized nature of convergent substitutions rather than poor alignment quality. Furthermore, the results of AU test did not reject the topology of this tree (Supplementary Table 1, *P* = 0.49). This result demonstrates that the same pattern of evolutionary event associated with morphological convergence occurred at both the phenotypic and sequence levels.

Our analysis detected multiple amino acid sites that had potentially been under morphological convergent evolution regarding the emergence of perforate SPC, in the candidate genes. The PCOC approach, which detects convergent evolution by modeling shifts in amino acid preference between ancestral and convergent phenotypes, has been successful in detecting the convergent evolution of sequence level in PEPC protein among plant samples (Rey et al. 2018). However, in our dataset, the number of PCOC sites was proportional to the total number of substitutions in each gene but not to the gene length, suggesting that PCOC analysis may include false positives (Supplementary Fig. 3a and b). Therefore, additional analysis was needed to conduct convergence site extraction with other criteria to detect the sites that might have more functional significance. Here, we further examined the entire gene sequence using the traditional method, which applies stricter criteria than PCOC. This method has been used to identify sites strongly suggestive of functional variation by detecting convergent substitutions specifically occurring on branches associated with phenotypic transitions (Zhang and Kumar 1997). Using these 2 approaches, we found that 72.5% of the sites detected by the traditional method were also detected by the PCOC method, which uses less stringent criteria for determining convergence (Supplementary Fig. 2). This suggests that the 2 methods employ different approaches, and that integrating both analyses enables more robust detection of convergent sites. A plausible explanation for the lower overlap between CSUBST and the traditional approach, compared with that between PCOC and the traditional approach, is the greater divergence time represented in our dataset relative to the benchmark datasets used in previous CSUBST studies (Kohler et al. 2015; Fukushima and Pollock 2023). Consistent with this interpretation, CSUBST analyses yielded *ω_C_* values below 1 for all candidate genes, with *dS_C_* generally comparable to or higher than *dN_C_* (Supplementary Table 4). These patterns suggest that synonymous-site changes may obscure codon-level convergence signals in these deeply diverged lineages, potentially reflecting substitutional saturation. Consequently, amino acid-based approaches would be more suitable than codon-based methods for detecting convergent substitutions in this dataset.

One gene, *spc33*, was consistently detected as a candidate gene in our phylogenetic analysis and, regardless of the approach used, showed a higher number of convergent substitutions than other genes. This gene is one of three known SPC-related genes, *spc14*, *spc18*, and *spc33* (van Peer et al. 2010; van Driel et al. 2008). van Peer et al. (2010) demonstrated that *spc33* is a transmembrane protein of SPCs by examining *Schizophyllum commune* (perforate type). The *spc33*-knockout mutant of *S. commune* exhibited the SPC loss, suggesting *spc33* is crucial for SPC formation. Given this evidence, combined with our findings, we conclude that *spc33* appears closely associated with the morphological convergence of SPC. Therefore, the gene that plays a role in SPC formation may also be involved in the evolution of SPC morphology. Furthermore, considering that BLAST analysis did not detect *spc33* from taxa other than perforate and imperforate species (Table 3), we propose *spc33* emerged just before the emergence of imperforate SPCs. This result supports Padamsee et al. (2012), who suggested that different genes other than *spc33* are involved in the SPC development in vesiculate species such as *Wallemia sebi*. Therefore, at this point, *spc33* is the promising candidate gene for morphological evolution of SPCs, and it is biologically valid to discuss the convergent evolution of SPC morphology using *spc33* as a starting point. We also found that the other candidate gene g6611.t1, which encodes SUMO, was in the top 5% regarding the proportion of convergent substitution sites (Fig. 3). Due to extensive functional roles of SUMO (Gupta et al. 2020), further analysis is essential to clarify its potential involvement in SPC morphological changes. SPCs are known to interact with multiple intracellular substances, such as the ER, cytoplasm, and cytoskeleton (Bracker and Butler 1964; Moore and Marchant 1972). Although we performed functional characterization of the candidate genes *in silico*, we did not find any functional association with SPC in our candidate genes except *spc33*. Future investigation into SPC-related intracellular components should be helpful in revealing functional links with remaining 6 genes.

We found that the specific SPC-associated convergent sites were located within amino acid sites 354–359, a short region of *spc33* (Fig. 4). This phenomenon led us to further question whether these substitutions have a functional role in morphological differentiation of SPCs. It is known that *spc33* is responsible for constructing SPCs (van Peer et al. 2010). Additionally, we determined that *spc33* evolved to the same pattern of amino acid sequences during perforate SPC evolution. These findings support our hypothesis that the SPC-forming protein SPC33 is involved in morphological differences of SPCs and morphological differentiation of perforate SPC is determined by a few key sites. Especially in the site 357. The nucleic acid difference at the third position of Punst (*Punctularia strigosozonata*), Glotr (*Gloeophyllum trabeum*), and Phaca (*Phanerochaete carnosa*) likely occurred after they acquired the CGN codon. This means that parallel evolution occurred in both nucleotide and amino acid sequences of *spc33* during morphological convergence of perforate SPCs. In other words, during the morphological convergence of perforate SPC, convergent evolution at the same site in the same gene from the same nucleotide residue was observed. Although further validation is needed since we cannot rule out the possibility that other genetic substrates participate in the emergence of perforate SPCs, here we say the same site-level substitutions can be involved in the morphological convergence of perforate SPCs. Therefore, the morphological difference between imperforate SPC and perforate SPC may be regulated by a limited number of key sites. If so, there may be few evolutionary paths leading to the change to perforate SPC.

In conclusion, we successfully provided suggestive evidence for the correlation of convergence between amino acid sequence evolution and the morphological evolution of perforate SPCs. Nucleotide and amino acid changes in the same sequence of the same gene corresponded to morphological convergence. We also showed that the present/absent pattern of *spc33* and the short region of *spc33* containing sites with perfect substitution pattern directly were linked with morphological evolution of SPC. These findings are the first step for clarifying the genetic basis of morphological evolution of SPC. The methods used also made the convergent substitution pattern detectable more easily. Our findings contribute to both clarifying the genetic basis of morphological convergence and demonstrating how genome-wide surveys can be used to elucidate fungal evolutionary morphology.

## Supplementary Material

jkag176_Supplementary_Data

## Data Availability

The genome data analyzed in this study are listed in Table 1. All scripts used in this study, the list of 2,560 single-copy genes along with their sequence files, and all raw and processed data from the traditional method, PCOC, CSUBST, and ESL-PSC analyses are available at https://github.com/toiizuka/SPC-convergence-pipeline. Multiple sequence alignments for all RBH datasets, phylogenetic trees, and output files from ancestral sequence reconstruction are available at Zenodo 10.5281/zenodo.20521591. Supplemental material available at G3 online.
