# Supplementary material for: Direct link between convergent evolution at sequence level and phenotypic level of septal pore cap in Agaricomycotina
Source: G3 (Bethesda). 2026 Jul 6;16(9):jkag176. doi: 10.1093/g3journal/jkag176 (PMC13535464; doi:10.1093/g3journal/jkag176)
Supplement: Supplementary file 1 [file jkag176_supplementary_data.zip › Supplementary_Figure_S1_G3-2026-406841.pdf]

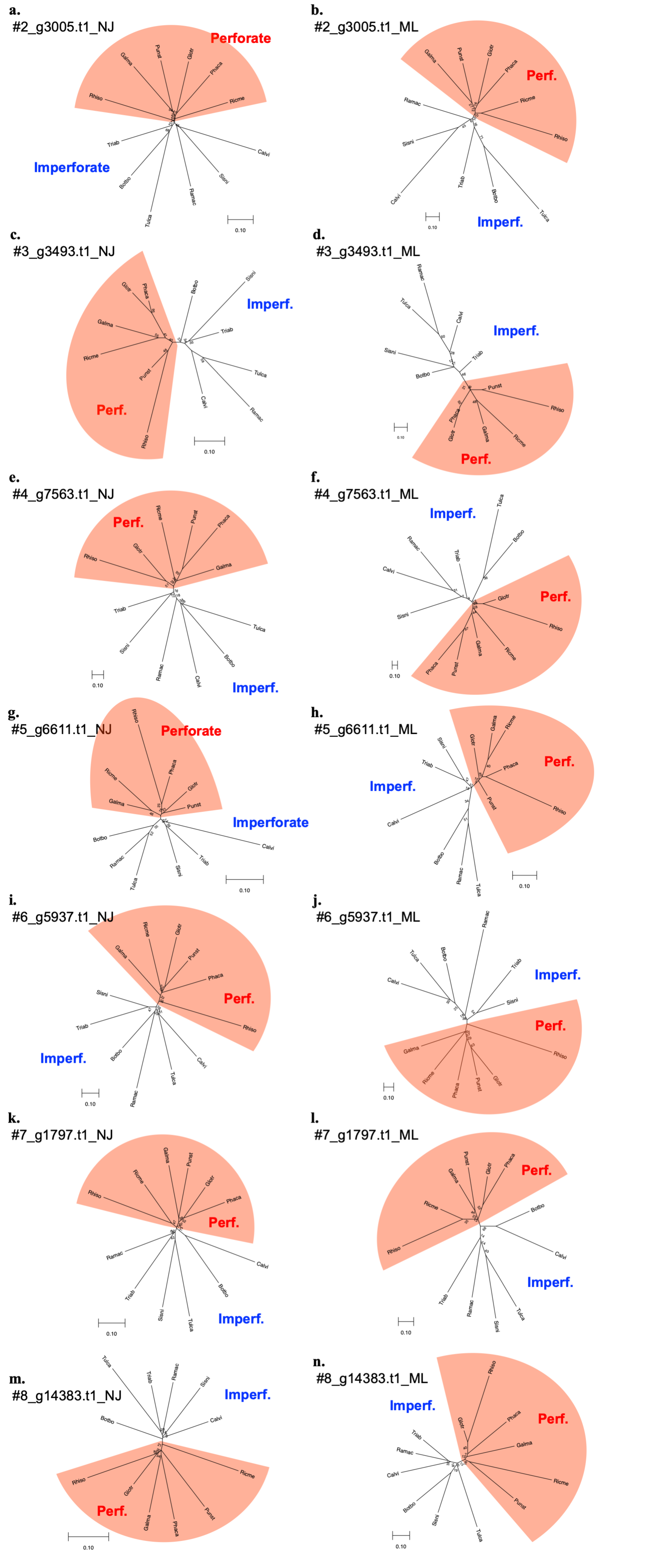

Supplementary Fig. S1. Phylogenetic relationships of correlated genes #2–#8 shown in Table 2. The gene trees were produced using the neighbor-joining (NJ) and maximum-likelihood (ML) methods.
