# Supplementary material for: Direct link between convergent evolution at sequence level and phenotypic level of septal pore cap in Agaricomycotina
Source: G3 (Bethesda). 2026 Jul 6;16(9):jkag176. doi: 10.1093/g3journal/jkag176 (PMC13535464; doi:10.1093/g3journal/jkag176)
Supplement: Supplementary file 1 [file jkag176_supplementary_data.zip › Supplementary_Figure_S3_G3-2026-406841.pdf]

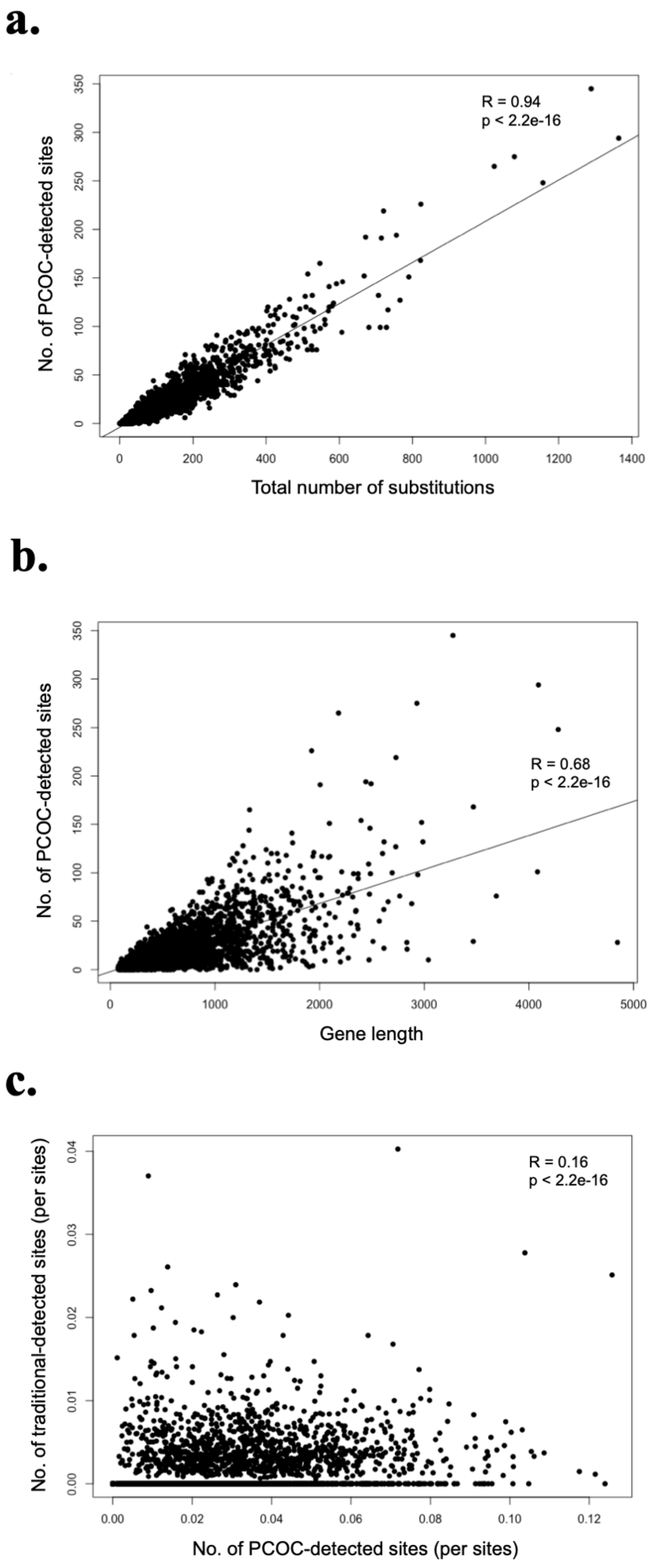

Supplementary Fig. S3. Regression analysis showing (a) relationship between the number of PCOC-detected sites (y-axis) and the total number of substitutions (x-axis), (b) no clear correlation between the number of PCOC-detected sites (y-axis) and gene length (x-axis), and (c) no correlation in the number of convergent substitutions per gene length between PCOC approach (x-axis) and traditional approach (y-axis). The black dots represent the correlation between the number of whole substitutions and PCOC sites for each gene. Black line indicates approximate straight line of the data.
