# Supplementary material for: Direct link between convergent evolution at sequence level and phenotypic level of septal pore cap in Agaricomycotina
Source: G3 (Bethesda). 2026 Jul 6;16(9):jkag176. doi: 10.1093/g3journal/jkag176 (PMC13535464; doi:10.1093/g3journal/jkag176)
Supplement: Supplementary file 1 [file jkag176_supplementary_data.zip › Supplementary_Figure_S4_G3-2026-406841.pdf]

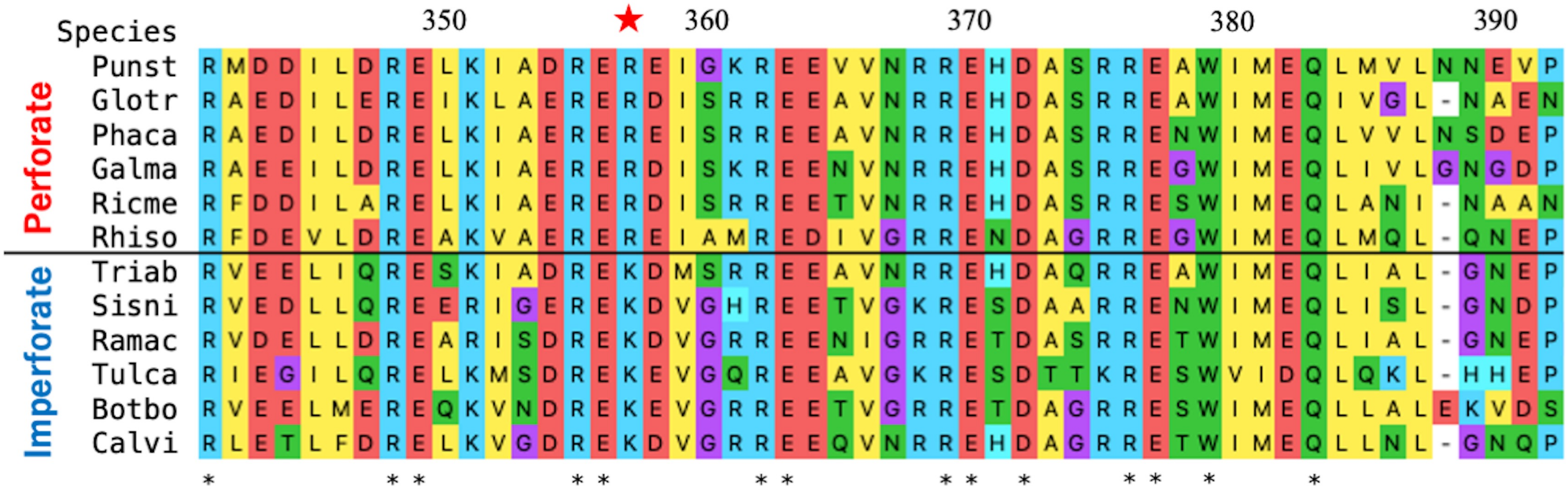

Supplementary Fig. S4. Multiple alignment of site 357 (red star) and neighboring sites in *spc33*. The abbreviations of the species names are as follows: Punst, *Punctularia strigosozonata*; Glotr, *Gloeophyllum trabeum*; Phaca, *Phanerochaete carnosa*; Galma, *Galerina marginata*; Ricme, *Rickenella mellea*; Rhiso, *Rhizoctonia solani* AG1-IB; Triab, *Trichaptum abietinum*; Sisni, *Sistotremastrum niveocremeum*; Ramac, *Ramaria rubella*; Tulca, *Tulasnella calospora*; Botbo, *Botryobasidium botryosum*; Calvi, *Calocera viscosa*.
