# Supplementary material for: Direct link between convergent evolution at sequence level and phenotypic level of septal pore cap in Agaricomycotina
Source: G3 (Bethesda). 2026 Jul 6;16(9):jkag176. doi: 10.1093/g3journal/jkag176 (PMC13535464; doi:10.1093/g3journal/jkag176)
Supplement: Supplementary file 1 [file jkag176_supplementary_data.zip › Supplementary_Figure_S5_G3-2026-406841.pdf]

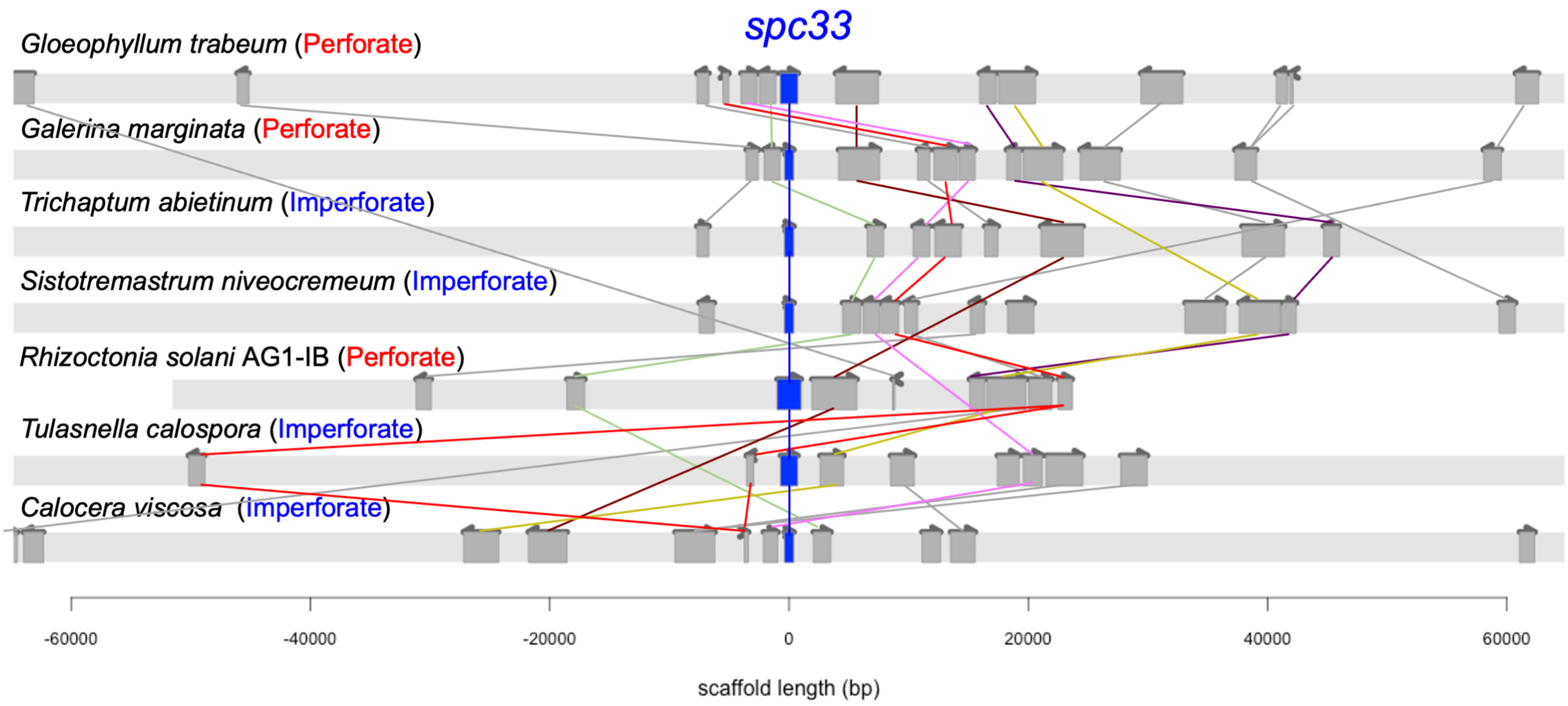

Supplementary Fig. S5. Conserved local synteny surrounding *spc33* across representative fungal species. Representative examples from seven species are shown, while orthologs were identified in at least 8 of the 13 analyzed species, including species not explicitly illustrated. Orthologous genes are connected by lines to indicate orthology relationships.
