# Supplementary material for: Direct link between convergent evolution at sequence level and phenotypic level of septal pore cap in Agaricomycotina
Source: G3 (Bethesda). 2026 Jul 6;16(9):jkag176. doi: 10.1093/g3journal/jkag176 (PMC13535464; doi:10.1093/g3journal/jkag176)
Supplement: Supplementary file 1 [file jkag176_supplementary_data.zip › Supplementary_Figure_S6_G3-2026-406841.pdf]

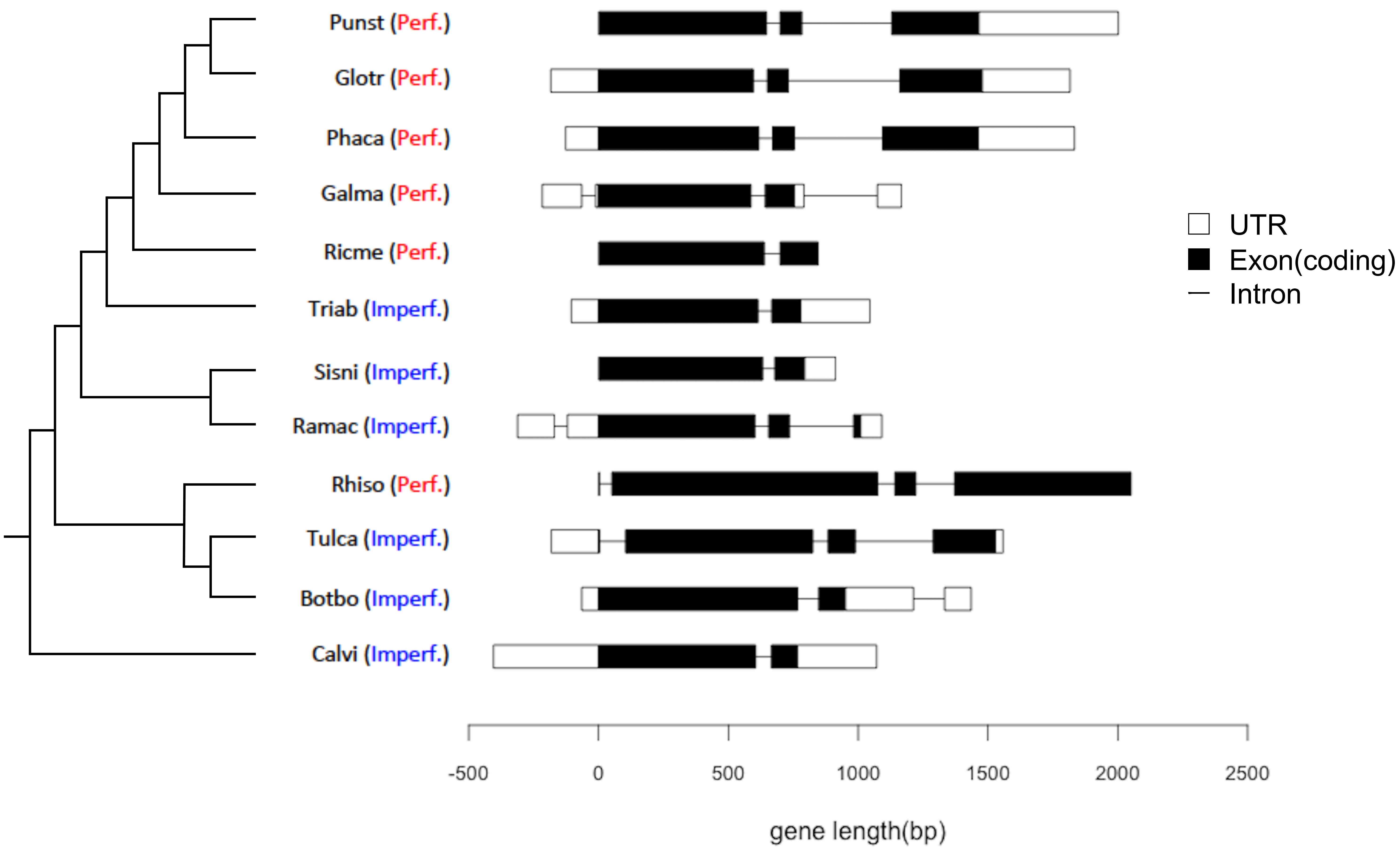

Supplementary Fig. S6. Exon–intron structures of *spc33* across representative fungal species with perforate (Perf.) and imperforate (Imperf.) SPC types. The presence of introns and the conservation of exon and intron number and length among closely related species indicate that the gene structure of *spc33* was established once and maintained through vertical inheritance, arguing against retrotransposon- or virus-mediated horizontal gene transfer. The abbreviations of the species names are as follows: Punst, *Punctularia strigosozonata*; Glotr, *Gloeophyllum trabeum*; Phaca, *Phanerochaete carnosa*; Galma, *Galerina marginata*; Ricme, *Rickenella mellea*; Triab, *Trichaptum abietinum*; Sisni, *Sistotremastrum niveocremeum*; Ramac, *Ramaria rubella*; Rhiso, *Rhizoctonia solani* AG1-IB; Tulca, *Tulasnella calospora*; Botbo, *Botryobasidium botryosum*; Calvi, *Calocera viscosa*.
